# Physical environmental and occupational factors inducing work-related neck and shoulder pains among self-employed tailors of informal sectors in Ethiopia, 2019: results from a community based cross-sectional study

**DOI:** 10.1186/s12889-020-09351-8

**Published:** 2020-08-20

**Authors:** Tesfaye Hambisa Mekonnen, Dawit Getachew Yenealem, Demiss Mulatu Geberu

**Affiliations:** 1 Department of Environmental and Occupational Health and Safety, Institute of Public Health, College of Medicine and Health Sciences, University of Gondar, P.O. Box 196, Gondar, Ethiopia; 2 Department of Health Systems and Policy, Institute of Public Health, College of Medicine and Health Sciences, University of Gondar, P.O. Box 196, Gondar, Ethiopia

**Keywords:** Neck and shoulder, Tailors, Self-employed, Ethiopia

## Abstract

**Background:**

Musculoskeletal disorders (MSD) caused by occupational-related factors continue to place huge burdens on global workforces. Significant numbers of workers report potential adverse health outcomes related to the condition, such as physical injury, disability, and decline in quality of life. Occupational-related MSD also poses additional burdens to healthcare services and diminishes productivity at work. The condition usually worsens in informal sectors where the work environments are often poorly designed. This paper explored occupational and physical environmental factors that induce work-related neck and/or shoulder pains among self-employed tailors in Gondar city, Northwest Ethiopia.

**Methods:**

We conducted across-sectional survey from April to May 2019 on 422 tailors selected with systematic random sampling technique. Nordic Musculoskeletal questionnaire was used to measure pains in neck and/or shoulder, and the questionnaire was pretested and administered by interviewers. Work-related factors such as working posture, rest break, training in safety and health, and the availability of adjustable chairs at workplaces were assessed. The significance of associations was set at a < 0.05 *p*-value and adjusted odds ratios (AOR) with a confidence interval (CI) of 95% were used to determine strength of associations.

**Results:**

A total of 419 tailors participated with a response rate of 99.3%. The mean age and mean years of experience were 29.23 (SD ± 7.03) and 1.48 (SD ± 0.50) years, respectively. The study found that the prevalence of pain in either neck or shoulder or both sites in the last 12 months was 66.6% (*N* = 279) [95% CI (62.1, 71.1)]. Pains in shoulder and neck were observed in 72.1% (*N* = 302)[95% CI (67.8, 76.4)] and 68.3% (*N* = 286) [95% CI (64.0, 72.6)] of the interviewees, respectively. The majority, 78.1% (*n* = 218) of those with pains indicated they were prevented from doing normal daily activities. Work experience (AOR = 1.81), rest break (AOR = 2.13), awkward working posture (AOR = 2.60), prolonged sitting (AOR = 2.00) and inadequate light (AOR = 5.02) were significantly associated factors of neck and/ or shoulder pains.

**Conclusion:**

Work-related neck and/or shoulder pain induced by physical factors of the work environment among self-employed tailors is pervasive in Ethiopia. Efforts to curb the condition, therefore, need to impalement diverse approaches addressing the physical environment and occupational factors. We also promote the integration of schemes for the effective use of rest breaks into health and safety programs in the workplace.

## Background

Musculoskeletal disorder (MSD) ensuing from exposure to various workplace factors is amongst the chronic public health challenges, and it substantially affects the global workforces. Many employees report potential adverse health outcomes related to the situation, such asphysical injury, disability [1–3] and decline in quality of life [1, 4, 5]. The 2016 Global Burden of Disease (GBD) report also indicates that musculoskeletal disorders were the leading cause of disability-adjusted life years (DALYs) (61.6%) [6], which is the second potential reason for years lived with disability [7]. Work-related upper extremity disorders, including neck and shoulder are well-recognized occupational diseases of the musculoskeletal systems, leading to considerable compensation claims [8, 9]. Survey of the Health and Safety Executive (HSE) showed that in 2017/2018,of the total number of days lost to work-related MSDs, work-related upper extremity disorders (WUEDs) accounted for the loss of estimated2.6 million days [10]. Moreover, studies have documented that 6–76% of the working population suffer from neck and shoulder pains annually [11, 12].

The study of musculoskeletal disorder and its social and economic consequences is mostly curbed to developed nations. Well documented data are deficient in developing countries, which makes the extent of its consequences difficult to estimate [13]. In developing countries, health and safety procedures are often disregarded, and infrastructures and systematic preventive approaches remain extremely slothful [14]. Consequently, only 5–10% of workers in developing nations including Ethiopia get access to basic occupational health services [15]. Non-compliance with and poor implementation of safety and health standards often worsen in informal sectors where typically vulnerable workforces, such as young workers, pregnant women, and the elderly appear to predominate in such countries [14].Tailoring/sewing shops/ are amongst the informal industries where numerous employees workin poorly designed workplaces in Ethiopia.

Tailors usually perform monotonous and repetitive jobs involving prolonged sitting [16] and frequent movements of the upper extremities [17, 18]. Scholars have demonstrated that upper extremity disorders such as shoulders and necks are prevalent in jobs performed in prolonged sitting positions [19, 20]. For instance,in a study in Iran, the prevalence of 49.7and 41.6% has been reported in neck and shoulder, respectively [21]. A study in India reported that 34% of tailors developed neck pains because of awkward postures associated with their jobs [22].

There have been a wide range of studies which support the considerable link between upper extremity impairments and socio-demographic factors, such as gender [3, 23, 24], age [11, 25, 26], marital status [21, 26], level of education, and experience [27]. Scholars have also proved that factors relating to the physical work environments,including working hours [21, 28], working posture [25, 28–30],shift work [26], rest break [30] as well as vibrations [31] importantly influence the experience of upper extremity disorders. Moreover, lifestyle factors, such as physical exercise [21, 25], alcohol use [25, 32] and smoking [33–35] and psychosocial-related factors including job dissatisfaction [36], job stress [30, 37], and time pressure [3] have been confirmed as potential risks for the development of musculoskeletal pains. However,in Ethiopia, studies involving tailors and the various adverse health outcomes they encounter because of the inherent nature of their jobs are inconclusive. Therefore, the current study estimated the prevalence and factors that induce work-related neck and/or shoulder musculoskeletal pains among self-employed tailor shop-owners of informal sectors in Gondar city, Ethiopia.

## Methods

### Study design, period and area

This study employed a cross-sectional design to explore the prevalence and occupation-related factors which induce upper extremity disorders (neck and/or shoulder pains) among self-employed tailors in Gondar city, Northwest Ethiopia, from April to May 2019. The city is one of the tourist destinations in the Amhara National Regional State, Northwest Ethiopia, 747 km from Addis Ababa, the capital of Ethiopia. It is located at latitude 12°36′N and longitude 37°28′E at an elevation of 2133 m above sea level. According to the 2007 Central Statistical Agency (CSA) of Ethiopia, the total population of the town was estimated at 323,900. The city is divided administratively into 12 sub-cities. There have been a total of 1300 tailors in the city during the data collection period.

### Sample size and populations

All self-employed tailors in Gondar city were the source population. Tailors who had worked for at least 12 months prior to the study were eligible for this study, while those witha history of injury, accidents, and pregnant women were excluded as they potentially confound the finding. We estimated the sample size using the single population proportion formula with the assumption of 50% prevalence of neck and/ or shoulder pains (as there was no previous evidence), 95% confidence interval (CI) and a 5% desired precision. After adding a 10% for non-response rate, 422 tailors were sampled using the systematic random sampling technique.

### Operational definitions

**Work-related upper extremity disorders**: self-reported pains, aches or discomforts in neck and shoulder anatomical body regions in the past 12 months.

**Perceived disability:** a pain disability point score of 3–6points [38].

**Perceived severity**: a pain intensity score of ≥50 or < 3 disability points [38].

**Awkward postures (AP)**: working with the neck bent without support, working with a bent wrist, working with the back bent without support, squatting and kneeling for two or more hours [39].

**Repetitive work (RW)**: work that involve repeating the same motion with less than 30 s or no variation every few seconds for two or more hours per day [39].

**Static postures (SP):** sitting or standing in a restricted space for two or more hours without changing positions per day [39].

**Job satisfaction**: generic job satisfaction scales core of 32 or above [40].

**Job stress**: workplace stress scale score of 21 or above [41].

**Body mass index (BMI)**: Self-report weight in kilograms divided by the square of height in meters (kg/m^2^) where (BMI = < 18.50 (underweight), BMI = 18.50–24.99 (normal), and BMI = ≥ 25.00 (overweight/obese).

**Physical exercise:** exercising any kind of sport activity at least two times per week for a duration of 30 min [42].

### Data collection tools and procedures

We employed a face-to-face interviewer- administered data collection technique. A standardized Nordic musculoskeletal questionnaire has been used to measure pains in necks and/ or shoulders [43]. The perceived satisfaction of tailors with their jobs was evaluated using a questionnaire on the 10-item generic job satisfaction scale [40]. The perceived job-related stress of workers was collected using the 8-item workplace stress scale questionnaire [41], whilethe 7-itemVon korff et al. questionnaire [38] was employed to evaluate the perceived severity and disability of pains. All the instruments have been used in previous studies carried out in the context of the country [32, 44]. Socio-demographic information such as gender, age, religion, educational status, marital status, income (as a monthly salary), and work experience was also collected. Factors related to the workplace, including working hours per day (< = 8 and > 8 per day), health and safety training (Yes/No), awkward work posture (working with bent/twist position without support, squatting and kneeling for two or more hours perday (Yes/No)), repetitive work (work that involves repeating same motion with < 30 s for two or more hours per day (Yes/No)), static work (sitting or standing in a restricted space for two or more hours without changing positions per day (Yes/No)), rest break (Yes/No), and physical work environment including adjustable chair designs (Yes/No), and adequacy of light at work (Yes/No) were assessed. Data regarding psychosocial factors, such as job satisfaction and job stress andindividual/behavioral factors such as any kind of physical exercise (Yes/No), frequency of physical exercise (two times and > two times per week for at least 30 min), alcohol use (Yes/No), smoking (Yes/No), and body mass index (a self-report weight in kilograms divided by squared height in meters) have also been gathered.

### Data quality control

Due emphasis has been given to the formulation of the data collection instrument. As such, the questionnaire was initially designed in English and translated to ‘Amharic’, the local language and back to English by two independent language experts. Seven trained data collectors (3 occupational health and safety professionals, 2 environmental health officers and 2 health informatics staffs) and two supervisors took part in the data collection. The data collectors and supervisors were adequately trained and oriented on issues related to the clarity of the questionnaire, objectives of the study, confidentiality of information, and voluntary participation (informed consent) of the participants. The principal investigator closely monitored the data collectors and supervisors. A pre-test was also conducted on 5% (19 participants) of the sample in Maksegnit town a week before the actual survey. Based on the pretest, the number of questions and some difficult words were changed.

### Methods of data analysis

Epi-info version 7.2.1.0 software program was used for data entry and Statistical Packages for Social Science (SPSS) version 20 software program for analysis. Percentages, frequency distributions and measures of central tendency were used to describe categorical variables. We checked the goodness of model fitness by Hosmer and Lemeshow (*p* = 0.087). The reliability of the standardized Nordic Musculoskeletal questionnaire was tested (Cronbach’s alpha = 0.82), and the reliability of the instrument was supposed tolerable [25]. The 10-item job satisfaction scale questionnaire was also checked for reliability and a Cronbach’s Alpha of 0.67 was found. Further, the 8-item job stress scale questionnaire was tested (Cronbach’s alpha = 0.81). The reliability of the Von korff et al. questionnaire employed for the assessment of perceived disability and severity of pains was checked (Cronbach’s alpha = 0.72). The instruments were, therefore, tolerable for their consistency in repeating what has been previously measured by the tools. Multicollinearity of the variables was checked by collinearity check using variable inflection factors (VIF < 5). The associations between the dependent and independent variables were examined by a binary logistic regression analysis. Independent variables at < 0.2*p*-values in the bivariable analysis were exported to a multivariable logistic regression model to further investigate the potential effects of confounders. Finally, we determined thelevel of statistical significance at ≤ 0.05 *p*-values and ascertained the strength of associations at adjusted odds ratio (AOR) with a confidence interval (CI) of 95%.

## Results

### Socio-demographic characteristics

In the current study, 419 self-employed tailors were participated with a response rate of 99.3%. The majority, 93.6% (*N* = 392) of the participants were males. Mean age was 29.23 (standard deviations (SD) ± 7.03) years, and 41.3% of respondents were in the age group of 30–39 years. Nearly one third, 36% (*N* = 150) of the tailors had completed primary school(1–8), while 9.1% (*N* = 38) indicated they had technical and vocational trainings. The majority, 52% (*N* = 218) of the tailors worked for < 5 years and the remaining for > 5 years with a mean of 1.48 (SD ± 0.50) years (Table 1).

**Table 1:** Socio-demographic characteristics of tailors, 2019, Ethiopia

Characteristic (*N* = 419)	Frequency (n)	Percent (%)
**Sex**		
Male	392	93.6
Female	27	6.4
**Age**		
< 29 years	152	36.3
30–39 years	173	41.3
> 40 years	94	22.4
**Marital status**		
Single	127	30.3
Married	258	61.6
Divorced/widowed/separated	34	8.1
**Religion**		
Orthodox	242	57.8
Muslim	126	30.1
Catholic	14	3.3
Protestant	37	8.8
**Educational Level**		
Can read and write	97	23.2
Primary school (1–8 grades)	150	35.8
Secondary school (9–12 grades)	134	32.0
Technical and vocational training	38	9.1
**Income (as a monthly salary)**		
< 5000 ETB	218	52.0
> 5000 ETB	201	48.0
**Work experience**		
≤ 5 years	218	52.0
> 5 years	201	48.0

Keys: *ETB* Ethiopian birr/currency, *1$US* 28 ETB, *N* number

### Individual /behavioral/and psychosocial characteristics

Of the participants, 79% (*N* = 331) described their body mass index (BMI) was normal, while 7.6% (*N* = 32) and 13.4% (*N* = 56) reported underweight and overweight, respectively. Out of the respondents, 69.7% (*N* = 292) of them stated they did not performphysical exercise, whereas 24.8% (*N* = 104) pointed out they exercised 1–2 and the rest > 3 times a week. Only 28.2% (*N* = 118) of the interviewees said they drank alcohol. Seventy-eight percent (*N* = 366) of the tailors showed they did not smoke, whereas 9.5 and 3.1% of them were past and current smokers, respectively. The study also indicated that 34.8% (*N* = 146) of the participants were chat chewers, of whom 31, 54 and 61% chewed every day, 1–3 times a week and occasionally, respectively. Regarding the psychosocial characteristics of the participants, 76% (*N* = 319) of them had good, 20.5% (*N* = 86) fair, and 3.3% (*N* = 14) poor relationships with their customers. About 37.5%(*N* = 157) of the tailors indicated they were satisfied and the remaining dissatisfied with their current jobs. More than half, 53.0% (*N* = 222) of the tailors reported their jobs were stressful.

### Work-related and physical environmentalcharacteristics

Table 2 shows the occupational and physical environment-related characteristics of the participants. The majority, 53% (*N* = 223) of the respondents described they worked for < 8 h a day. With regard to working days per week, 77 % (*N* = 323) of the respondents delineated they worked for < 7 days a week, and the rest for 7 days. Nearly half, 51.3% (*N* = 215) illustrated that their activities involved awkward working posture (bending/twisting for more than 2 h) a day. Regarding workplace illumination, 58.9% (*N* = 247) of the respondents presented there was no adequate light.

**Table 2:** Work-related and physical environmental characteristics, 2019, Ethiopia

Characteristic(***N*** = 419)	Frequency (N)	Percent (%)
**Working hours per day**		
< =8 h	223	53.2
> 8 h	196	46.8
**Working days per week**		
< 7 days	323	77.1
7 days	96	22.9
**Use of rest break**		
Yes	88	21
No	331	79
**Safety /ergonomic training**		
Yes	66	15.8
No	353	84.2
**Availability of adjustable chair**		
Yes	21	5.0
No	398	95.0
**Awkward working posture**		
Yes	215	51.3
No	204	48.7
**Hours spent sitting position per day**		
< =8 h	211	50.4
> 8 h	208	49.6
**Involving in repetitive work posture**		
Yes	266	63.5%
No	153	36.5%
**Comfortable work environment**		
Yes	133	31.7
No	286	68.3
**Adequate lightavailable at work**		
Yes	172	41.1
No	247	58.9

### Prevalence and characteristics of musculoskeletal pains

**Fig. 1:**
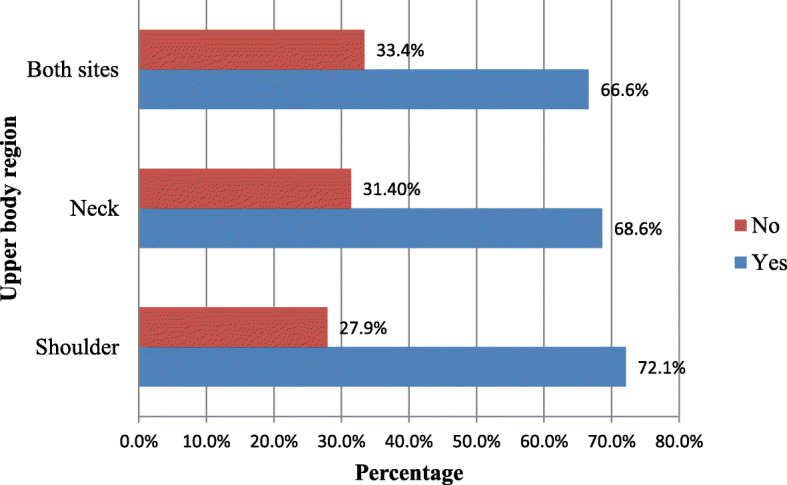
Prevalence of neck and shoulder pains in the past 12 months (*N* = 419)

This study revealed that the 12 month prevalence of painin either neckor shoulder or both sites was 66.6% (*N* = 279) [95% CI (62.1, 71.1)], and that of the last 7 days and 30 days was 33.2% (*N* = 139) [95% CI (28.3, 37.7)] and 42.2% (*N* = 177) [95% CI (37.5, 47.0)], respectively. Figure 1 represents the prevalence of pains in either neck or shoulder or both during the last 12 months. There was no difference in prevalence between male and female respondents (Pearson’s chi square (X^2^) = 0.186; *p*-value = 0.421). The majority, 44.1% (*n* = 123) of the participants who had neck and/ or shoulder pain was in theage group of 30–39 years. The prevalence of neck and/ or shoulder pain among the participants with < 5 years of employment duration was higher (58.1%) than those with > 5 years of work experience counterparts (X^2^ = 12.20; *p*-value < 0.0001). Visits to physicians for treatments of neck and/ or shoulder pain was reported by 22.6% (*n* = 63) of the victims. Sixty-two percent (*n* = 173) of the pain sufferers perceived their pain was severe, while 51.3% (*n* = 143) of them described it was a disabling.

### Occupational characteristics of musculoskeletal pains

About 30.5% (*n* = 85) of the respondents indicated they had pains for 1–7 days, 23.3% (*n* = 65) for 8–30 days, 32.0% (*n* = 89) for over 30 days but not every day, and 14.3% (*n* = 40) every day during the last 12 months. The study also found that 78.1% (*n* = 218) of the neck and shoulder pain sustainers were prevented from doing normal work at home or away from home during the last 12 months, of those 22.0% (*n* = 61), 37.3% (*n* = 104), 22.6% (*n* = 63) and 18.3% (*n* = 51) for 0 days, 1–7 days, 8–30 days and > 30 days, respectively (X^2^ = 15.34;*p* = 0.0001). Only a few, 17.9% (*n* = 50) of the participants with neck and/or shoulder pains appeared that they had trainings in safety/ergonomics (X^2^ = 2.96; *p* = 0.085). Moreover, of neck and/or shoulder pain victims, 57.0% (*n* = 159) of them said they sat for > 8 h a day and the rest for < 8 h and the difference was significant (X^2^ = 18.03; *p* = 0.0001). Out of the neck and/or shoulder cases, 74.6% (*n* = 208) of them reported they did not use rest break at work. The lighting condition at work in relation to those with pain was also assessed and over three fourths (*n* = 206) said they worked in tailoring shops with no adequate light and the difference was observed to be significant (X^2^ = 76.45; *p* < 0.0001). The majority (71.3%) of the victims were assessed to be involved in awkward/bending and twisting/ working postures for > 2 h a day with a more remarkable increase in neck and/or shoulder pains than those who worked in such postures for < 2 h a day (X^2^ = 23.04; *p* < 0.0001).

### Associated factors of neck and/or shoulder pains

Age, alcohol, educational level, work experience, lack of rest break, absence of sufficient light at work, working posture, prolonged sitting, and working hours per week were detected significant factors ofneck and/ or shoulder disorders in the bivariable analysis. The multivariable regression analysis showed that work experience, rest break, working posture, prolonged sitting, and absence of sufficient light at work remained significant risk factors for neck and/or shoulder pains.

Self-employed tailors who had worked for < 5 years were 1.92 times more likely to be at risk for neck and/orshoulder pains than their counterparts (> 5 years) [AOR = 1.92; 95% CI (1.11, 3.33)]. Participants who did not use rest breaks were 2.13 times at a higher probability of developing neck and/or shoulder pains than those who did [AOR = 2.13; 95% CI (1.20, 4.22)]. The odds of experiencing neck and/or shoulder pains increased by a factor of 2.60 times among tailors who usually worked in awkward /bending and/or twisting/ postures than those who did not [AOR = 2.60; 95% CI (1.45, 4.47)]. Those who usually worked in tailoring shops with inadequate light were 5.02 times more likely to face neck and/or shoulder pains than those who were engaged in shops with adequate light [AOR = 5.02; 95% CI (3.50, 9.03)]. The multivariable logistic regression analysis also yielded that tailors who usually performed their tasks in a prolonged sitting position were twice more likely to develop neck and/or shoulder pains than those who did not work in such positions [AOR = 2.00; 95% CI (1.20, 3.41)] (Table 3).

**Table 3:** Factors affecting work-related neck and/or shoulder pain among tailors, 2019, Ethiopia

C Characteristic (*N* = 419)	Neck and/or shoulder pain		COR(95% CI)	AOR(95% C.I)	*P*-value
	Yes (%)	No (%)			
**Age**					
≤ 29 years	93(33.3)	59(42.1)	1	1	
30–39 years	123(44.1)	50(35.7)	1.56(0.98,2.50)	1.14(0.35,2.01)	0.071*
> 40 years	63(22.6)	31(22.1)	1.29 (0.75,2.21)	1.03(0.01,1.07)	0.063*
**Alcohol use**					
Yes	87(31.2)	31 (22.1)	1.59(0.99,2.56)	1.23(0.15,1.97)	0.081*
No	192 (68.8)	109(77.9)	1		
**Educational level**					
Can read and write	62(22.2)	35(25.0)	0.55(0.23,1.29)	0.46(0.12,1.09)	0.470*
Primary school (1–8 grades)	94(33.7)	56(40.0)	0.52(0.23,1.18)	0.44(0.17,1.01)	0.614*
Secondary school (9–12 grades)	94(33.7)	40(28.6)	0.73(0.32,1.68)	0.51(0.21,1.03)	0.083*
Technical and vocational training	29(10.4)	9(6.4)	1	1	
**Work experience**					
≤ 5 year	162(58.1)	56(40.0)	2.08 (1.37,3.14)	1.92(1.11, 3.33)	0.001^+^
> 5 years	117(41.9)	84(60.0)	1	1	
**Hours spent sitting position/day**					
< 8 h/day	120(43.0)	91(65.0)	1	1	
> 8 h/day	159(57.0)	49(35.0)	2.46(1.62,3.75)	2.00(1.20,3.41)	0.001^+^
**Working days per week**					
< 7 days	207(74.2)	116(82.9)	1	1	
7 days	72(25.8)	24(17.1)	0.64(0.38,1.10)	0.47(0.16,1.01)	0.133*
**Rest break**					
Yes	71(25.4)	17 (12.1)	1	1	
No	208(74.6)	123 (87.9)	2.46 (1.33,4.55)	2.13(1.20,4.22)	0.0001^+^
**Adequate light at work**					
Yes	73 (26.2)	99 (70.7)	1	1	
No	206 (73.8)	41 (29.3)	6.81(4.34,10.70)	5.02(3.50,9.03)	001^+^
**Frequently bending/twisting**					
Yes	199 (71.3)	64 (45.7)	2.95(1.94,4.50)	2.60(1.45,4.47)	0001^+^
No	80 (28.7)	76 (54.3)	1	1	

Keys: -* = Significant in the bivariable analysis; ^+^ = Significant in the multivariable analysis, *N* Number, *AOR* Adjusted odds ratio, *CI* Confidence interval, *COR* Crude odds ratio

## Discussion

Work-related musculoskeletal pains are common in tailoring shop industries and are of occupational and public health important. Therefore, in order to devise effective preventive approaches, it is essential to analyzethe degreeof the problem and its occupational correlations with specific site of the body. This study explored the prevalence and associated factors of work-related neck and/or shoulder pains among self-employed tailors in Gondar city, Northwest Ethiopia. The finding of this study showed that the prevalence of musculoskeletal pains in either neck or shoulder or both (comorbid) among tailors during the last 12 months was 66.6%.The result also revealed that pains in shoulder and neck (separately) were observed in 72.1 and 68.3% of the participants, respectively. Self-employed workers, particularly of informal sectors, including tailors are not covered by labor laws and regulations in Ethiopia. As a result, labor inspection staffs are not authorized to legitimately provide information, technical support and advice on health and safety implementations. This is likely to result in increased prevalence of neck and shoulder pains among self-employed workers in the country. Our survey found a lower prevalence of neck and or shoulder pains compared to a study in India (91 and 88% in neck and shoulder,respectively) [45]. However, our finding is higher than that of 49.7% neck and 41.6% shoulder pain prevalence in study in Iran [21] and 31.67 and 38.33% prevalence of pain in neck and shoulder, respectively, from the report in India [46]. In Bangladesh, 23.7% of pain in neck has been documented [47]. These differences could be due to discrepancies in the methods used for case definitions (self-report symptoms versus clinical diagnosis), socio-economic characteristics, perception of pain and the culture of reporting perceived health conditions.

Our analysis demonstrated that the duration of employment was asignificant risk factor for increased prevalence of neck and shoulder pains. Accordingly, tailors with a shorter length of employment were more likely to experience work-related musculoskeletal pains than those with longer employment duration. This result substantiates previous data in Bangladesh [47] and Finland [48]. The investigation in Bangladesh has interpreted the finding in relation to the lack of exposure to workplace interactions and experience in meeting ergonomic hazards. According to the study in Finland, upper back pains, including neck and shoulder were more likely to increasein the newly employed workers because of a lack of acclimatization to the work environment, particularly to a high workload. Conversely, other investigations have shown that a longer duration of employment increases the risk of neck and shoulder pains [27, 45, 49]. This disagreement may be due to variations in the designs of the studies (prospective follow up versus cross-sectional), study populations (self-employed versus employed by others) and the nature of the work environments (informal tailor shops versus registered garment factories). Another possible explanation for this result may be that informal industries (also known as small and micro enterprises) in Ethiopia have been rising incredibly over the last decade. This is because the government of Ethiopia has introduced several reforms, such as the establishment of microfinance institutions, to provide access to loans, particularly to the poorest, thereby creating job opportunities for the citizens [50].

Consistent with literature [51], working posture (bending and/or twisting) has been identified as a considerable risk factor for neck and shoulder pains in the current investigation. A continuous deviation from normal/recommended working positions (usually neutral)/ could adversely affect neck and shoulder [52]. There have been wide documents which support the significant relations of working in prolonged sitting positions and the likelihood of developing neck and shoulder impairments [53–55]. A prospective study discovered thatworkers who involve in tasks performed with sitting for more than 95% of their working time were at increased risk of experiencing neck pains [54]. This could be related to the effects of the load exerted on neck muscles in a continual exposure to static working postures.

Our analysis has confirmed the results of several studies [56, 57] that a reasonable rest break was a protective factor for neck and shoulder pains. It has been explained that rest break allows stressed muscles and tissues to get recovery from pains and discomfort [56] by disrupting or decreasing exposure to repetitive or monotonous workloads and durations. Literature reveals that a temporary detachment from work with a given frequency, duration and type of work breaks (passive or active) plays a remarkable role in reducing the onset and proliferation of pains in the musculoskeletal systems [58]. Investigators have also clarified that the type of rest breaks (micro-pauses for 5–15 s every 10 min for relaxation exercises) and (macro-pause for 5 min every hour for stretching exercises), which are equally important, are recommended to be incorporated in to work schedules [59].

Our analysis also found that stressful physical work environment, such as the lack of sufficient light at work was associated remarkably with neck and shoulder pains. An investigation in the United Kingdom (UK) corroborates this result [60]. Self-employed workersin Ethiopia usually work insmall shops with poorly equipped physical (illumination) and ergonomic (chairs) infrastructures. Even, due to financial constraints, the majority usuallywork in their own small rooms. As such,natural light coming from outside that can provide sufficient viewsas an alternative is often skimpy. Consequently, tailors possibly adopt poor physical working postures, such as bending their necks forward and twisting sideways, which are the most common biomechanical risk factors for the experience of musculoskeletal pains.

This study is perhaps the first in Ethiopia to examine physical working conditions which induce work-related musculoskeletal disorders among self-employed tailors. Various adverse health outcomes linked to poor physical working environments inself-employed workers of informal sectors are often under recognized in the country. Consequently, the result of this study is essential to initiate steps towards measures necessary to prevent informal employees from those health conditions. However, given that the data obtained were retrospective and self-reported, recall bias might be anticipated, perhaps under mining the findings of the study. Moreover, the analysis of physical working postures which is important to estimate the degree to which the workers have been exposed to ergonomic hazards was not performed. A validated survey questionnaire was used to minimize this limitation. Further, because we conducted the studyonly on the informal sector of self-employed workers, it may be difficultto generalize the results to workers of other economic sectors. We recommend future research need toinclude adequate sample sizes from various economic sectors and to use prospective epidemiological designs to conduct such study.

## Conclusion

Work-related neck and/or shoulder pain induced by physical factors of the work environment among self-employed tailors is pervasive in Ethiopia. Work experience, working posture, prolonged sitting, lack of adequate light and rest break were risk factors associated with neck and shoulder pain. Efforts to curb the conditions, therefore, need to impalement diverse approaches addressing the physical environment and occupational factors. We also promote the integration of schemes for the effective use of rest breaks into health and safety programs in the workplace.

## Data Availability

The datasets used and/or analyzed during the current study are available from the corresponding author on reasonable request.

## Abbreviations

AOR: Adjusted Odd Ratio
BMI: Body Mass Index
CI: Confidence Interval
COR: Crude Odd Ratio
DALYs: Disability adjusted life years
ETB: Ethiopia Birr
GBD: Global burden of diseases
HSE: Health and Safety Executive
MSDs: Musculoskeletal Disorders
SD: Standard deviations
SPSS: Statistical Package for Social Science
UK: The United Kingdom
USA: The United States of America
WMSDs: Work-related Musculoskeletal Disorders
WUEDs: Work-related upper extremity disorders
